# High prevalence of unawareness of HCV infection status among both HCV-seronegative and seropositive people living with human immunodeficiency virus in Taiwan

**DOI:** 10.1371/journal.pone.0251158

**Published:** 2021-05-06

**Authors:** Chun-Yuan Lee, Pei-Hua Wu, Meng-Wei Lu, Tun-Chieh Chen, Po-Liang Lu

**Affiliations:** 1 Department of Internal Medicine, Kaohsiung Municipal Siaogang Hospital, Kaohsiung, Taiwan; 2 Graduate Institute of Medicine, Kaohsiung Medical University, Kaohsiung, Taiwan; 3 Department of Medicine, College of Medicine, Kaohsiung Medical University, Kaohsiung, Taiwan; 4 Division of Infectious Diseases, Department of Internal Medicine, Kaohsiung Medical University Hospital, Kaohsiung, Taiwan; 5 Department of Public Health, College of Health Science, Kaohsiung Medical University, Kaohsiung, Taiwan; 6 Infection Control Office, Kaohsiung Municipal Ta-Tung Hospital, Kaohsiung, Taiwan; 7 Department of Laboratory Medicine, Kaohsiung Medical University Hospital, Kaohsiung, Taiwan; 8 Liquid Biopsy and Cohort Research Center, Kaohsiung Medical University, Kaohsiung, Taiwan; University of New Mexico Health Sciences Center, UNITED STATES

## Abstract

**Objectives:**

HCV infection status awareness is crucial in the HCV care continuum for both HCV-seropositive (HCV-positive status awareness) and seronegative (HCV-negative status awareness) populations. However, trends in the unawareness of HCV infection status (UoHCV) remain unknown in HIV-positive patients. This study investigated UoHCV prevalence, the associated factors of UoHCV, and its association with HCV-related knowledge in HIV-positive patients.

**Methods:**

For this cross-sectional, multicenter, questionnaire-based study, 844 HIV-infected participants were recruited from three hospitals in Taiwan from June 2018 to March 2020. Participants were grouped by HCV serostatus (HCV-seronegative [n = 734] and HCV-seropositive [n = 110]) and categorized by their HIV diagnosis date (before 2008, 2008–2013, and 2014–2020). Exploratory factor analysis was used to categorize the 15 items of HCV-related knowledge into three domains: route of HCV transmission, HCV course and complications, and HCV treatment.

**Results:**

The prevalence of UoHCV was 58.7%–62.6% and 15.1%–31.3% in the HCV-seronegative and HCV-seropositive groups, respectively, across 3 periods. More participants with UoHCV believed that HCV infection was only contracted by intravenous injection. In the HCV-seropositive group, participants with UoHCV were more likely to have HIV diagnosis before 2008 (vs. 2014–2020), be men who have sex with men (vs. people who inject drugs), and have hepatitis A virus seronegativity. In the HCV-seronegative group, participants with UoHCV were more likely to have a recent history of sexually transmitted diseases, but had a lower education level, had received less information on HCV infection from clinicians, and were less likely to have heard of HCV infection prior to the research. UoHCV was associated with lower scores for three domains of HCV-related knowledge in both groups.

**Conclusions:**

The negative association of UoHCV with HCV-related knowledge suggests that strategies targeting patients according to their HCV serostatus should be implemented to reduce UoHCV and eradicate HCV infection among HIV-positive patients.

## Introduction

The global seroprevalence of the hepatitis C virus (HCV) is approximately 2.5% [1]. Although treatment with direct-acting antivirals (DAAs) can lead to the elimination of HCV viremia and a curative outcome in more than 90% of patients with chronic HCV infection [2], several barriers to eradicating HCV infection still exist, including the high costs of drugs [3], frequent loss to follow-up after diagnosis [4], high rate of early HCV reinfection among patients who have recently received drug injections [5], and ongoing high-risk behaviors associated with HCV infection (even after clearance of HCV infection) [6]. The identification of undiagnosed patients, timely provision of DAAs to HCV-seropositive populations [7, 8], and the prevention of transmission among at-risk HCV-seronegative populations must be prioritized to eliminate HCV infection [7].

Awareness of HCV infection status is crucial for both HCV- seropositive and HCV-seronegative populations. Although the short- and long-term impacts of HCV-positive status awareness among HCV-seropositive patients on their risk behavior remain matters of debate [9–12], HCV-positive status awareness is essential in the HCV care continuum in terms of treatment eligibility and taking medical advice on viral transmission [12, 13]. However, unawareness of HCV infection status (UoHCV) remains common in the HCV-seropositive population, with prevalence rates of 20.5% in Italy [14], 16.6%–35.1% in Taiwan [15–17], and 14%–51% in the United States [18, 19]. In the HCV-seronegative population at risk of contracting HCV, people who inject drugs (PWID) may engage in high-risk behaviors (e.g., sharing a syringe or injecting themselves with drugs) less frequently if they are aware of their HCV infection status (i.e., HCV-negative status awareness) [9, 12]. However, a nationwide screening program in Taiwan revealed a 33% prevalence of UoHCV in the HCV-seronegative population [16].

People living with human immunodeficiency virus (HIV) infection (PLWH) are at risk of HCV infection because the transmission routes of HCV infection, such as unprotected sex and drug injection, are similar to those of HIV infection [6, 20, 21]. Moreover, individuals coinfected with HCV and HIV are less likely to seek HCV care [22–24], which may contribute to a significantly decreased quality of life and quicker progression of liver disease, especially in those who are homeless or marginally housed [25]. Additionally, patients with HCV/HIV coinfection have higher rates of death and disease progression, including the progression of histological fibrosis/cirrhosis and decompensated liver disease, than do patients with HCV monoinfection [26]. Therefore, HCV screening, treatment, and prevention strategies should be strictly implemented among PLWH [27]. However, although strategies have been implemented worldwide to combat the spread of HCV, no study has explored the prevalence or associated factors of UoHCV among PLWH [11]. Serostatus awareness facilitates the next step in the continuum of HCV care, namely providing affected patients with access to health care, relevant consultation, and potential treatment, which are necessary to eradicate HCV. Furthermore, knowledge regarding HCV infection is essential for the further utilization of HCV treatment [28, 29]. In one study, the majority of participants had limited knowledge regarding the complications of chronic HCV infection despite being aware of the high prevalence of HCV among men who have sex with men (MSM) [30]. However, little is known regarding the association of UoHCV with knowledge of HCV among PLWH. We hypothesized that UoHCV is negatively associated with an individual’s HCV-related knowledge, regardless of their HCV serostatus.

We conducted a cross-sectional questionnaire-based study at three HIV referral centers in Taiwan from June 2018 to March 2020. We evaluated the prevalence of UoHCV, explored the determinants of UoHCV, and evaluated the associations of UoHCV with different domains of HCV-related knowledge (i.e., route of HCV transmission, HCV course and complications, and HCV treatment) among a sample of PLWH stratified by HCV serostatus.

## Materials and methods

### Study design and setting

This cross-sectional, multicenter, questionnaire-based study was conducted from June 1, 2018, to March 31, 2020, at Kaohsiung Municipal Siaogang Hospital and Kaohsiung Municipal Ta-Tung Hospital, which are regional hospitals in southern Taiwan, and at Kaohsiung Medical University Hospital, which is the largest referral center for PLWH in southern Taiwan. The HCV seropositivity in southern Taiwan is 8.6% [31], which is higher than that in northern Taiwan (1.2%–2.7%) [32].

### Development of the study questionnaire

A questionnaire was designed to investigate participants’ awareness of HCV infection status, knowledge of different aspects of the disease, perceived risk of HCV infection, and assessment of potential exposure to HCV.

An expert group comprising an HIV case manager, HIV specialists, hepatologists, and researchers developed the preliminary questionnaire used in this study. The preliminary questionnaire was then modified based on feedback from 30 PLWH after they had completed a pretest. The questionnaire was tested again with 20 PLWH and further modified. Because of the lack of a standardized scoring system for evaluating different categories of HCV-related knowledge, the questionnaire items were modified after a review of pertinent studies [28–30, 33]. The section on the perceived risk of HCV infection was also modified [34].

The final questionnaire comprised variables in the following five categories: sociodemographic characteristics, awareness of HCV infection status, knowledge of HCV infection, perceived risk of HCV infection, and assessment of potential exposure to HCV (S1 File). Participants were instructed to answer the 15 HCV-related knowledge items by providing one of the following responses: “yes,” “no,” or “I do not know” [35]. One point was awarded for each correct response, and no point was awarded for incorrect or “I do not know” responses. Therefore, the mean scale scores ranged from 0 to 1, with higher scores indicating greater HCV-related knowledge. Variables correlated with the respondents’ perceived risk of HCV infection were measured on a 5-point Likert scale (strongly disagree, disagree, neither agree nor disagree, agree, and strongly agree).

### Participants and study procedure

Two trained investigators screened PLWH by reviewing their available medical records at the participating hospitals for the period from January 1, 2000, to March 31, 2020. Patients who were less than 20 years old during the screening period (June 1, 2018, to March 31, 2020), had not undergone HCV antibody tests within 1 year before enrollment, or were lost to follow-up during the screening period were excluded. The participants completed the questionnaires on Google Forms.

The participants were classified into one of two groups according to their HCV serostatus: a HCV-seronegative group and a HCV-seropositive group. They were then stratified according to their awareness of their HCV infection status. Finally, each of the two groups was further divided into two subgroups: the unawareness/HCV-seronegative (subgroup 1), awareness/HCV-seronegative (subgroup 2), unawareness/HCV-seropositive (subgroup 3), and awareness/HCV-seropositive (subgroup 4).

The study was approved by the Institutional Review Board of Kaohsiung Medical University Hospital (KMUHIRB-SV(I)-20180024) and adhered to the principles of the Declaration of Helsinki. The investigators obtained signed informed consent forms from all patients before enrollment.

### Definitions

The participants were categorized by three distinct periods based on the calendar year of their first confirmed HIV diagnosis: before 2008 (period 1, before the remission of the HIV epidemic among PWID) [36], 2008–2013 (period 2, remission of the HIV epidemic among PWID but before the introduction of oral DAAs), and 2014–2020 (period 3, after the introduction of oral DAAs).

In this study, awareness of HCV infection status was defined as participants’ self-reported recognition of their HCV infection status at the time of enrollment in the study (i.e., HCV-seropositive patients’ awareness of their HCV-positive status and HCV-seronegative patients’ awareness of their HCV-negative status), whereas UoHCV was defined as participants’ self-reported unawareness of their HCV infection status [8, 16].

The behavioral indicators of a high risk of exposure to HCV infection were modified from other studies and included using any intravenous recreational drugs [12], engaging in chemosexual behaviors within the preceding 6 months [37], having a sexual partner within the preceding 6 months (assessment options were no sexual partners, one regular sexual partner, no regular sexual partners/less than five partners, and no regular sexual partner/more than five partners) [37], and engaging in other activities involving sexual contact within the preceding 6 months [37].

### Outcomes of interest

The primary outcome of interest was the prevalence of UoHCV across the three study periods, stratified by the participants’ HCV serostatus. Secondary outcomes were factors associated with UoHCV and the associations of UoHCV with the mean scores for three domains of HCV knowledge (route of HCV transmission, HCV course and complications, and HCV treatment) among the participants stratified by their HCV serostatus.

### Statistical analysis

Descriptive analyses were performed on the characteristics of the participants in the four subgroups. The categorical and continuous variables in each subgroup were compared through χ^2^ tests (or Fisher’s exact tests) and independent *t* tests, respectively. The prevalence of UoHCV was defined as the proportion of participants with UoHCV at the time of questionnaire completion. The trend analyses of the prevalence of UoHCV stratified by HCV serostatus in the three periods were performed using a Cochran–Armitage trend test with modified ridit scores.

Backward stepwise binary logistic regressions were performed to calculate odds ratios and evaluate associations in the bivariate and multivariable analyses between surveyed factors and UoHCV among all the participants and among those in the two HCV serostatus groups. To simultaneously consider the effects of all variables in the multivariable model, we adopted a backward approach.

To determine the validity of the 15 items used to measure the participants’ knowledge regarding HCV infection, an item analysis was performed for the assessment of item discrimination. We also performed exploratory factor analysis by using principal axis factoring with varimax rotation to investigate the structural domain of the 15 items, and three domains were finally categorized: route of HCV transmission (domain 1), HCV course and complications (domain 2), and HCV treatment (domain 3). Cronbach’s α was used to measure the internal consistency of the items in each structural domain, where α represented the function of the number of items in a test. Cronbach’s α ≥ 0.7 indicates high reliability.

Finally, to determine the association of UoHCV with the means of the total and domain-specific scores of HCV-related knowledge, we employed a multilinear regression model with a backward approach. *β* along with 95% confidence intervals were calculated to estimate the effects of UoHCV and directions of all associations. A backward approach was also adopted to enable the effects of all the variables to be simultaneously considered in the multivariable model.

All tests were two-tailed, and p < 0.05 was considered significant. Statistical analyses were performed using SPSS Statistics version 25.0 (IBM, Armonk, NY, USA).

## Results

### Participants

**Fig 1:**
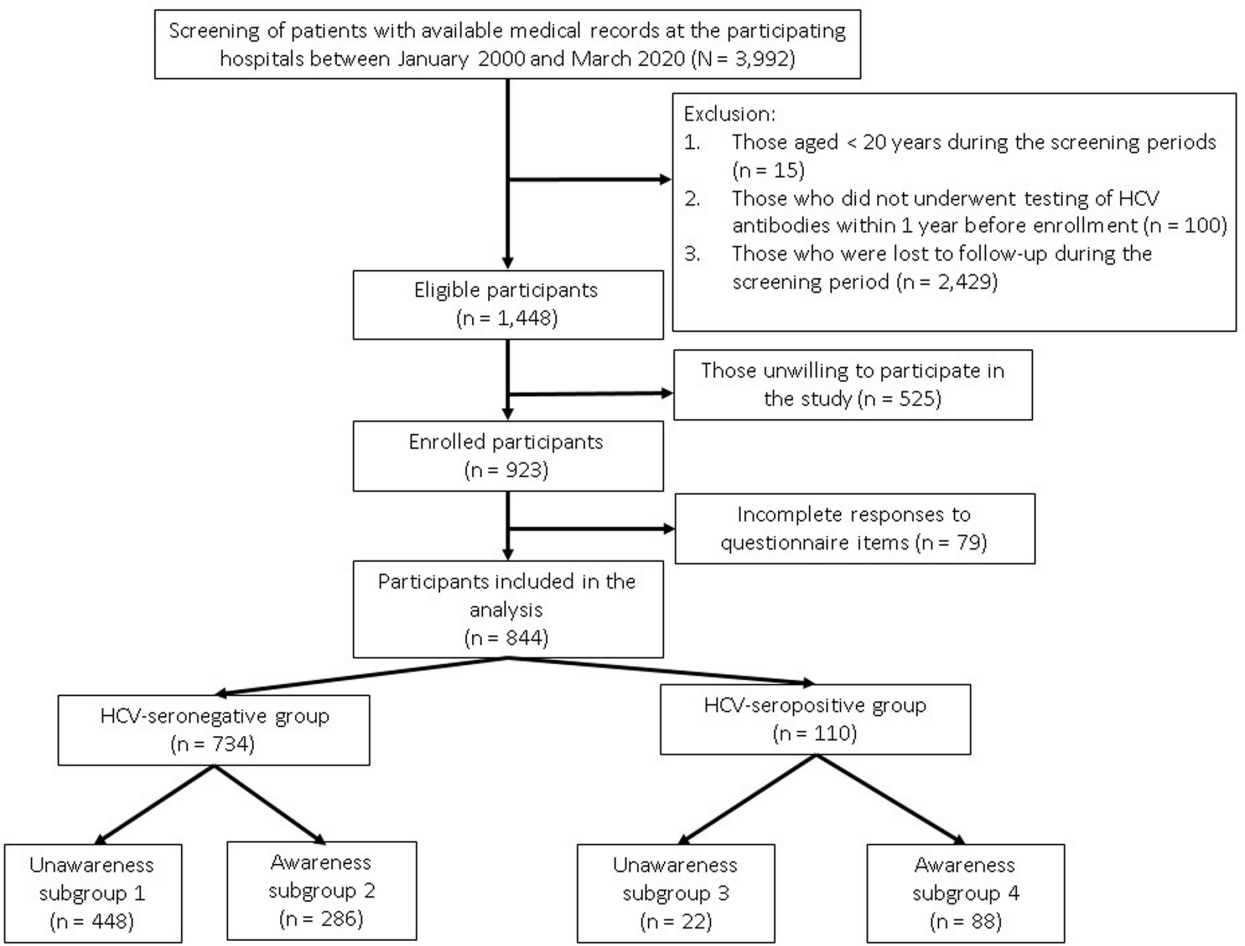
Study flowchart.

The study flowchart is displayed in Fig 1. Among the 1448 eligible PLWH, 525 were unwilling to participate in the study, and 79 were excluded for incomplete responses to questionnaire items. A total of 844 PLWH were included in the final analysis. They were divided into HCV-seronegative (n = 734) and HCV-seropositive (n = 110) groups. The two groups were further divided into subgroups 1 (unawareness/HCV-seronegative; n = 448), 2 (awareness/HCV-seronegative; n = 286), 3 (unawareness/HCV-seropositive; n = 22), and 4 (awareness/HCV-seropositive; n = 88).

### Characteristics of the study participants

Table 1 presents the sociodemographic characteristics, laboratory profiles, perceived risk of HCV infection, and high-risk behaviors for HCV infection of the participants in each subgroup. The mean (standard deviation) age at enrollment among all the participants was 36.6 (±9.8) years, and 98.1% of the participants were men. The routes of HIV transmission included men who have sex with men (MSM) (73.5%), bisexual contact (13.0%), heterosexual contact (8.3%), and drug injection (5.2%). Approximately 21.0%, 30.2%, and 48.8% of the participants were diagnosed as having HIV in periods 1, 2, and 3, respectively.

**Table 1:** Comparison of sociodemographic characteristics of 844 PLWH between those with and without awareness of their HCV infection status, stratified by HCV serostatus.

		*All N = 844*	*HCV-seronegative group*		*P*	*HCV-seropositive group*		*P*
			*Subgroup 1 (Unawareness)*	*Subgroup 2 (Awareness)*		*Subgroup 3 (Unawareness)*	*Subgroup 4 (Awareness)*	
			*N = 448*	*N = 286*		*N = 22*	*N = 88*	
**Sociodemographic variables**								
• Period of HIV diagnosis, n (%)					0.621			0.167
	Period 1 (before 2008)	177 (21.0)	75 (16.7)	49 (17.1)		8 (36.4)	45 (51.1)	
	Period 2 (2008–2013)	255 (30.2)	135 (30.1)	95 (33.2)		4 (18.2)	21 (23.9)	
	Period 3 (2014–2020)	412 (48.8)	238 (53.1)	142 (49.7)		10 (45.5)	22 (25.0)	
• Male gender, n (%)		828 (98.1)	443 (98.9)	281 (98.3)	0.471	21 (95.5)	83 (94.3)	>0.999
• Age		36.6 (9.8)	35.3 (9.8)	36.6 (9.3)	0.091	38.3 (10.8)	42.5 (9.4)	0.70
• Education above college level, n (%)		452 (53.6)	226 (50.4)	193 (67.5)	<0.001	7 (31.8)	26 (29.5)	0.835
• Employment, n (%)		690 (81.8)	360 (80.4)	250 (87.4)	0.013	15 (68.2)	65 (73.9)	0.592
• Marriage, n (%)		42 (5.0)	21 (4.7)	13 (4.5)	0.389	2 (9.1)	6 (6.8)	0.403
• HIV diagnosis in Kaoping area, n (%)		762 (90.3)	400 (89.3)	258 (90.2)	0.689	20 (90.9)	84 (95.5)	0.345
• HIV-related risk assessment					0.720			0.023
	MSM	620 (73.5)	339 (75.7)	223 (78.0)		15 (68.2)	43 (48.9)	
	Heterosexual	70 (8.3)	42 (9.4)	20 (7.0)		2 (9.1)	6 (6.8)	
	Bisexual	110 (13.0)	65 (14.5)	42 (14.7)		2 (9.1)	1 (1.1)	
	PWID	44 (5.2)	2 (0.4)	1 (0.3)		3 (13.6)	38 (43.2)	
• History of sexually-transmitted diseases within the preceding 6 months, n (%)		150 (17.8)	94 (21.0)	40 (14.0)	0.017	5 (22.7)	11 (12.5)	0.307
• Has your doctor ever provide you the information about your HCV infection status before		369 (43.7)	95 (21.2)	193 (67.5)	<0.001	10 (45.5)	71 (80.7)	0.001
• Have ever heard of HCV		678 (80.3)	299 (66.7)	273 (95.5)	<0.001	18 (81.8)	88 (100.0)	<0.001
**Laboratory data at time of questionnaire, n (%)**								
• HAV Ab seropositivity		538 (64.0)	276 (61.7)	187 (65.6)	0.290	11 (50.0)	64 (74.4)	0.027
• HBs Ag seropositivity		84 (10.0)	45 (10.0)	26 (9.1)	0.681	2 (9.1)	11 (12.6)	0.646
**Perceived risk of HCV infection**								
• Only those who inject medication intravenously can get hepatitis C		2.12 (1.00)	2.3 (1.02)	1.86 (0.88)	<0.001	2.18 (1.30)	2.03 (0.99)	0.558
• The sexual behavior styles that I like put me at risk of hepatitis C infection.		2.95 (1.09)	2.99 (0.99)	2.91 (1.23)	0.347	3.23 (1.11)	2.81 (1.12)	0.118
• I am more worried about hepatitis C virus than HIV		2.90 (1.04)	2.90 (0.97)	2.90 (1.16)	0.952	2.64 (1.00)	3.00 (0.98)	0.125
**Assessment of potential exposures to HCV within the preceding 6 months**								
• Use of intravenous form of recreational drugs, n (%)					0.126			0.215
	No use	756 (89.6)	412 (92.0)	274 (95.8)		18 (81.8)	52 (59.1)	
	Yes, less than a year	35 (4.1)	23 (5.1)	5 (1.7)		1 (4.5)	6 (6.8)	
	Yes, 1–3 years	20 (2.4)	10 (2.2)	5 (1.7)		1 (4.5)	4 (4.5)	
	Yes, more than 3 years	33 (3.9)	3 (0.7)	2 (0.7)		2 (9.1)	26 (29.5)	
• Engagement in chemosexual behaviors within the preceding 6 months, n (%)		109 (12.9)	65 (14.5)	34 (11.9)	0.311	5 (22.7)	5 (5.7)	0.013
• Status of having a sexual partner within the preceding 6 months, n (%)					0.383			0.832
	No sexual partner	316 (37.4)	164 (36.6)	91 (31.8)		12 (54.5)	49 (55.7)	
	Regular sexual partner	334 (39.6)	173 (38.6)	125 (43.7)		7 (31.8)	29 (33.0)	
	No regular sexual partners, less than 5 partners	144 (17.1)	79 (17.6)	54 (18.9)		3 (13.6)	8 (9.1)	
	No regular sexual partner, more than 5 partners	50 (5.9)	32 (7.1)	16 (5.6)		0 (0.0)	2 (2.3)	
• Sexual experiences within the preceding 6 months, n (%)								
	Ever experience sadomasochism	23 (2.7)	10 (2.2)	9 (3.1)	0.447	1 (4.5)	3 (3.4)	>0.999
	Ever experience group sex participation	82 (9.7)	50 (11.2)	27 (9.4)	0.458	1 (4.5)	4 (4.5)	>0.999
	Ever experience Insertive/receptive unprotected anal intercourse	440 (52.1)	236 (52.7)	172 (60.1)	0.047	9 (40.9)	23 (26.1)	0.195
	Ever experience vaginal sex	87 (10.3)	42 (9.4)	32 (11.2)	0.426	2 (9.1)	11 (12.5)	>0.999

Abbreviations: Ab, antibody; Ag, antigen; HBs, hepatitis B surface; HCV, hepatitis C virus; HIV, human immunodeficiency virus; PWID, people who inject drugs; MSM, men who have sex with men; PLWH, people living with human immunodeficiency virus.

### Trend analysis of the prevalence of UoHCV across the three periods stratified by participants’ HCV serostatus

**Fig 2:**
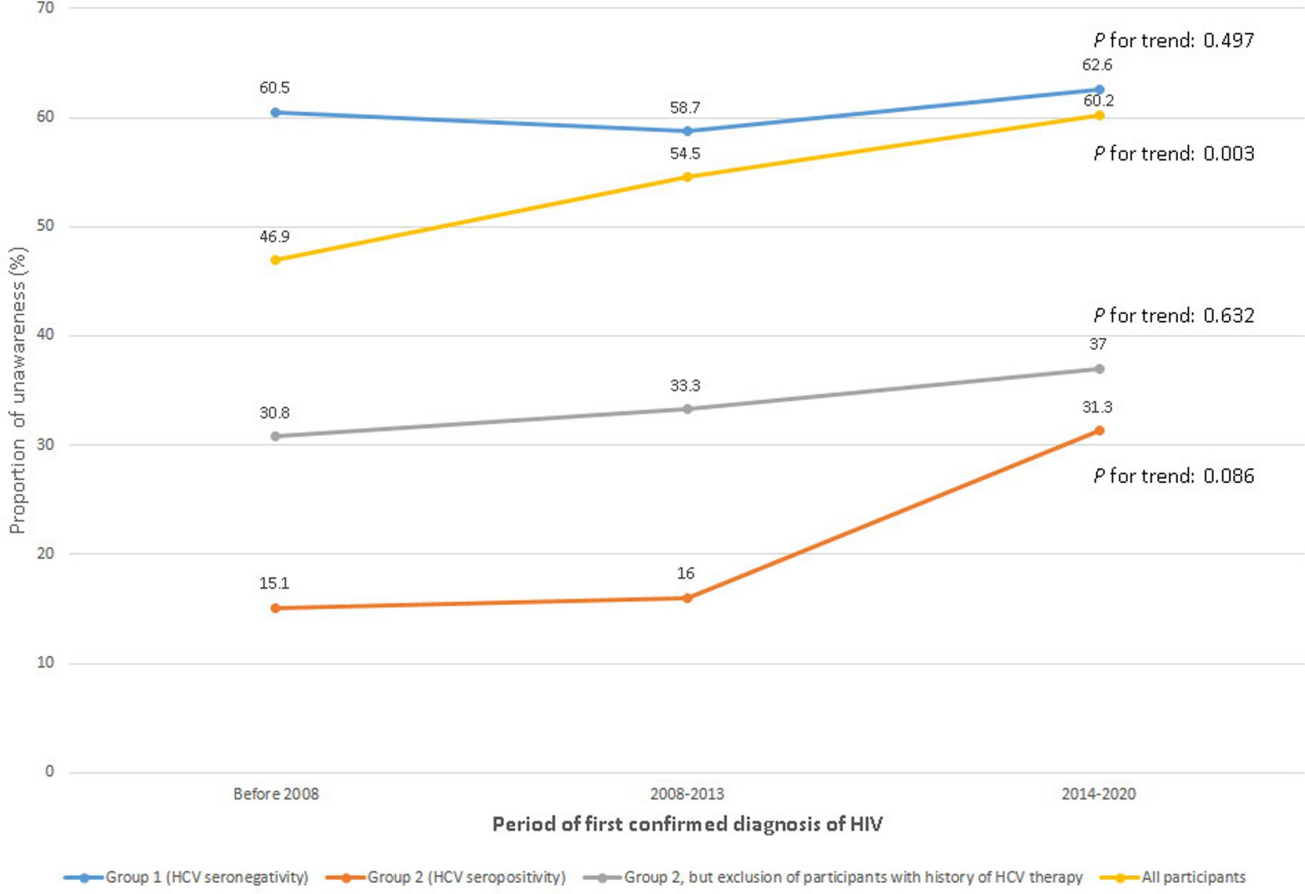
Trend analyses of prevalence of UoHCV stratified by HCV serostatus in three periods according to the calendar year of first confirmed HIV diagnosis (period 1 [before 2008], period 2 [2008–2013], and period 3 [2014–2020]). Prevalence of unawareness of HCV infection status across the three periods among all participants and among HCV-seronegative individuals, HCV-seropositive individuals receiving HCV therapy, and HCV-seropositive individuals not receiving HCV therapy. Cochran–Armitage trend test with modified ridit scores was used to analyze the trends in the prevalence of unawareness of HCV infection status for periods 1 to 3. Abbreviations: HCV, hepatitis C virus.

The prevalence rate of UoHCV was 46.9% in period 1, 54.5% in period 2, and 60.2% in period 3 (p for trend = 0.003; Fig 2). The overall prevalence of UoHCV was 58.7%–62.6% in the HCV-seronegative group (p for trend = 0.497) and 15.1%–31.3% in the HCV-seropositive group (p for trend = 0.086). Participants with a history of treatment for HCV were excluded from the HCV-seropositive group because all of these patients were aware of their HCV infection status and were thus not part of the target population; this exclusion increased the prevalence of UoHCV to 33.8% (ranging from 30.8% in period 1 to 37.0% in period 3, p for trend = 0.632).

### Factors associated with UoHCV among PLWH stratified by HCV serostatus

After stratification by HCV serostatus, the two groups differed in terms of factors associated with UoHCV in a binary logistic regression (Table 2). In the HCV-seropositive group, the proportion of participants who believed that intravenous injection was a requirement for contracting HCV was greater among individuals with UoHCV than among those who were aware of their HCV status. Furthermore, these individuals with UoHCV were less likely to have received an HIV diagnosis in period 3 (vs. period 1), be PWID (vs. MSM), and have hepatitis A virus (HAV) seropositivity, compared with individuals with awareness of their HCV status. In the HCV-seronegative group, compared with the participants who were aware of their HCV status, those with UoHCV were more likely to have a history of sexually transmitted diseases within the preceding 6 months and believe that intravenous injection was a requirement for contracting HCV. Moreover, they were less likely to have received higher education, previously heard of HCV infection, and received information on HCV infection from clinicians (Table 2).

**Table 2:** Bivariate and multivariable analyses of factors associated with UoHCV among PLWH stratified into two groups: HCV-seronegative group (n = 734) and HCV-seropositive group (n = 110).

			*All participants*			*HCV-seronegative group*		*HCV-seropositive group*	
			*% of UoHCV*	*Bivariate analysis*, *crude OR (95% CI)*	*Multivariable analysis*, *adjusted OR (95% CI)*	*% of UoHCV*	*Multivariable analysis*, *adjusted OR (95% CI)*	*% of UoHCV*	*Multivariable analysis*, *adjusted OR (95% CI)*
**Sociodemographic variables**									
• Period of HIV diagnosis									
	Period 1 (before 2008)		46.9	Reference		60.5		15.1	Reference
	Period 2 (2008–2013)		54.5	1.357 (0.924–1.994)		58.7		16.0	
	Period 3 (2014–2020)		60.2	1.713 (1.201–2.443)**		62.6		31.3	0.029 (0.001–0.918)*
• Gender									
	Female		37.5	Reference		50.0		16.7	
	Male		56.0	2.125 (0.765–5.900)		61.2		20.2	
• Age, per 1-year increase			N/A	0.974 (0.961–0.988)***		N/A		N/A	
• Education above college level									
	No		60.5	Reference	Reference	70.5	Reference	19.5	
	Yes		51.5	0.696 (0.529–0.915)**	0.481 (0.334–0.692)***	53.9	0.462 (0.317–0.673)***	21.2	
• Employment									
	No		61.7	Reference		71.0		23.3	
	Yes		54.3	0.739 (0.517–1.057)		59.0		18.8	
• Marriage									
	No		57.2	Reference		61.3		22.2	
	Yes		39.4	0.269 (0.135–0.535)***		57.1		13.8	
• HIV diagnosis in Kaoping area									
	No		61.0	Reference		63.2		33.3	
	Yes		55.1	0.786 (0.493–1.253)		60.8		19.2	
• HIV-related risk assessment									
	MSM		57.1	Reference		60.3		25.9	Reference
	Heterosexual		62.9	0.204 (0.107–0.391)***		67.7		25.0	
	Bisexual		60.9	0.254 (0.145–0.447)***		60.7		66.7	
	PWID		11.4	0.625 (0.393–0.997)*		66.7		7.3	0.028 (0.001–0.877)*
• History of sexually-transmitted diseases within the preceding 6 months									
	No		53.5	Reference	Reference	59.0	Reference	18.1	
	Yes		66.0	1.690 (1.168–2.445)**	2.012 (1.261–3.212)**	70.1	2.190 (1.349–3.554)**	31.3	
• Has your doctor ever provide you the information about your HCV infection status before									
	No		76.8	Reference	Reference	79.1	Reference	41.4	
	Yes		28.5	0.120 (0.088–0.164)***	0.167 (0.118–0.236)***	33.0	0.154 (0.107–0.222)***	12.3	
• Have ever heard of HCV									
	No		92.2	Reference	Reference	92.0	Reference	100.0	
	Yes		46.8	0.075 (0.042–0.134)***	0.151 (0.081–0.283)***	52.3	0.158 (0.085–0.297)***	17.0	
**Laboratory data at time of questionnaire, n (%)**									
• HAV Ab seropositivity									
	No		60.3	Reference		63.6		33.3	Reference
	Yes		3.3	0.754 (0.566–1.004)		59.6		14.7	0.015 (0.001–0.270)**
• HBs Ag seropositivity									
	No		55.8	Reference		60.9		20.8	
	Yes		56.0	1.006 (0.636–1.584)		63.4		15.4	
• HCV Ab seropositivity									
	No		61.0	Reference	Reference	N/A		N/A	
	Yes		20.0	0.160 (0.098–0.261)***	0.428 (0.229–0.800)**	N/A		N/A	
**Perceived risk of HCV infection**									
• Only those who inject medication intravenously can get hepatitis C			N/A	1.527 (1.317–1.771)***	1.405 (1.162–1.699)***	N/A	1.402 (1.147–1.714)**	N/A	4.912 (1.130–21.358)*
• The sexual behavior styles that I like put me at risk of hepatitis C infection			N/A	1.099 (0.970–1.245)		N/A		N/A	
• I am more worried about hepatitis C than HIV			N/A	0.964 (0.845–1.098)		N/A		N/A	
**Assessment of potential exposures to HCV**									
• Use of intravenous form of recreational drugs									
	No use		56.9	Reference		60.1		25.7	
	Yes, less than a year		68.6	1.654 (0.799–3.426)		82.1		14.3	
	Yes, 1–3 years		55.0	0.927 (0.380–2.262)		66.7		20.0	
	Yes, more than 3 years		15.2	0.135 (0.052–0.354)		60.0		7.1	
• Engagement in chemosexual behaviors within the preceding 6 months									
	No		54.4	Reference		60.3		17.0	
	Yes		64.2	1.502 (0.990–2.282)		65.7		50.0	
• Status of having a sexual partner within the preceding 6 months									
	No sexual partner		55.7	Reference		64.3		19.7	
	Regular sexual partner		53.9	0.930 (0.683–1.267)		58.1		19.4	
	No regular sexual partners, less than 5 partners		56.9	1.052 (0.707–1.566)		59.4		27.3	
	No regular sexual partner, more than 5 partners		64.0	1.414 (0.762–2.625)		66.7		0.0	
• Sexual experiences within the preceding 6 months									
	Ever experience sadomasochism								
		No	55.9	Reference		61.3		19.8	
		Yes	47.8	0.723 (0.315–1.657)		52.6		25.0	
	Ever experience group sex participation								
		No	55.0	Reference		60.6		20.0	
		Yes	62.2	1.347 (0.843–2.152)		64.9		20.0	
	Ever experience Insertive/receptive unprotected anal intercourse								
		No	55.7	Reference		65.0		16.7	
		Yes	55.7	1.000 (0.762–1.312)		57.8		28.1	
	Ever experience vaginal sex								
		No	56.3	Reference		61.5		20.6	
		Yes	50.6	0.795 (0.510–1.240)		56.8		15.4	

Note: *p < 0.05;

**p < 0.01;

***p < 0.001.

Abbreviations: Ab, antibody; Ag, antigen; HBs, hepatitis B surface; HCV, hepatitis C virus; HIV, human immunodeficiency virus; PWID, people who inject drugs; MSM, men who have sex with men; N/A, not available; PLWH, people living with human immunodeficiency virus; UoHCV, unawareness of HCV infection status.

### Development of the HCV knowledge scale among PLWH

The item analysis initially indicated that the original 15 items for measuring HCV knowledge exhibited satisfactory discriminant validity. Exploratory factor analysis finally identified three domains of the 15 items: route of HCV transmission (domain 1), HCV course and complications (domain 2), and HCV treatment (domain 3). Cronbach’s α revealed suitable reliability in the three domains, which were thus further analyzed (S1 Table).

### Association between UoHCV and HCV-related knowledge scores stratified by HCV serostatus

Table 3 presents the correct response rates to the 15 HCV-related knowledge items among PLWH with and without awareness of their HCV status, stratified by HCV serostatus. Overall, the correct response rates to the questions ranged from 16.8% to 71.1%. The following three questions had the lowest rates of correct responses: “can hepatitis C virus infection be prevented by vaccines?” (16.8%), “does hepatitis C infection commonly not result in any symptoms?” (31.3%), and “does the successful treatment of hepatitis C virus infection prevent reinfection?” (39.3%). The question “can hepatitis C virus infection be treated?” had the highest proportion of correct responses (71.1%).

**Table 3:** Comparison of correct responses to structural questions on HCV knowledge between participants with and without awareness of their HCV status, stratified by HCV serostatus.

		*Correct answer*	*HCV-seronegative group*		*P-value*	*HCV-seropositive group*		*P-value*
			*Unawareness*	*Awareness*		*Unawareness*	*Awareness*	
			*N = 448*	*N = 286*		*N = 22*	*N = 88*	
Route of HCV transmission								
	Does hepatitis C virus can be transmitted through the blood?	Yes	49.6%	82.2%	<0.001	68.2%	85.2%	0.064
	Does hepatitis C virus can be transmitted through sexual behaviors?	Yes	41.7%	73.8%	<0.001	59.1%	75.0%	0.138
	Does hepatitis C virus can be transmitted through mother-to-child vertical transmission?	Yes	41.1%	69.6%	<0.001	45.5%	69.3%	0.036
	Are the infection routes of HIV similar to those of the hepatitis C virus?	Yes	41.7%	72.7%	<0.001	59.1%	72.7%	0.212
	During sexual behaviors, does mucosa hemorrhage of sexual contact parts due to excessive intensity makes hepatitis C virus infection easier?	Yes	40.3%	76.2%	<0.001	57.9%	68.4%	0.386
	Is blood the major transmission routes of hepatitis C virus?	Yes	38.8%	61.5%	<0.001	54.5%	71.6%	0.125
Course and complication of HCV								
	If you are infected with HIV, does this mean you are more likely to be infected with hepatitis C virus?	Yes	38.6%	67.5%	<0.001	72.7%	64.8%	0.480
	Does the successful treatment of hepatitis C virus infection prevent reinfection?	No	31.5%	47.2%	<0.001	40.9%	53.4%	0.294
	Hepatitis C virus mostly cures itself, and no treatment is needed	No	51.6%	75.2%	<0.001	63.6%	87.5%	0.008
	Does Hepatitis C infection commonly not result in any symptoms?	Yes	21.4%	42.3%	<0.001	27.3%	46.6%	0.101
	Do complications after hepatitis C virus infection include cirrhosis and liver cancer?	Yes	45.5%	78.0%	<0.001	59.1%	84.1%	0.01
	Does HIV increase complication probability after hepatitis C virus infection (such as cirrhosis and liver cancer)?	Yes	40.6%	66.8%	<0.001	45.5%	69.3%	0.036
Treatment of HCV								
	Can hepatitis C virus infection be prevented by vaccines?	No	9.2%	21.7%	<0.001	31.8%	36.4%	0.690
	Can hepatitis C virus infection be treated?	Yes	57.8%	83.9%	<0.001	68.2%	97.7%	<0.001
	Can Hepatitis C virus infection be cured?	Yes	33.9%	53.5%	<0.001	40.9%	83.0%	<0.001

Abbreviations: HCV, hepatitis C virus; HIV, human immunodeficiency virus.

Multilinear regression analyses revealed that UoHCV was associated with lower mean scores overall and for each domain of HCV-related knowledge compared with HCV status awareness, both in the HCV-seronegative group and the HCV-seropositive group (Table 4).

**Table 4:** Association of UoHCV with HCV knowledge scores, stratified by knowledge domain in a multivariable linear regression^†^.

	*Total score*	*Domain 1 (Route of HCV transmission)*	*Domain 2 (HCV course and complications)*	*Domain 3 (HCV treatment)*
	*Adjusted Model Estimate (β)(95% CI)*	*Adjusted Model Estimate (β)(95% CI)*	*Adjusted Model Estimate (β)(95% CI)*	*Adjusted Model Estimate (β)(95% CI)*
Total participants				
• Unawareness of HCV infection status	-0.232 (-0.271–-0.192)***	-0.271 (-0.321–-0.220)***	-0.217 (-0.262–-0.173)***	-0.190 (-0.232–-0.147)***
HCV-seronegative group				
• Unawareness of HCV infection status	-0.239 (-0.281–-0.197)***	-0.283 (-0.337–-0.228)***	-0.222 (-0.270–-0.175)***	-0.182 (-0.227–-0.137)***
HCV-seropositive group				
• Unawareness of HCV infection status	-0.195 (-0.305–-0.084)**	-0.189 (-0.337–-0.040)*	-0.177 (-0.301–-0.054)**	-0.282 (-0.404–-0.160)***

Note: *p < 0.05;

**p < 0.01;

***p < 0.001.

^†^Adjustments were made in the multilinear regression for the period since participants received their HIV diagnosis and for their gender, age, education level, employment status, marital status, site of HIV diagnosis, HIV risk factors, history of sexually transmitted diseases in the preceding 6 months, and history of HCV therapy.

Abbreviations: Ab, antibody; CI, confidence interval; HCV, hepatitis C virus.

## Discussion

To the best of our knowledge, ours is the first study to reveal differences in the prevalence rates of UoHCV among PLWH; these rates were 61.0% in the HCV-seronegative group and 20.0% in the HCV-seropositive group. The prevalence rate in the HCV-seropositive group increased to 33.8% after the exclusion of participants with a history of HCV treatment, all of whom knew their HCV status. After stratification by HCV serostatus, the two groups differed in terms of their sociodemographic characteristics and laboratory variables associated with UoHCV, indicating the need for strategies to be tailored according to HCV serostatus when attempting to reduce UoHCV among PLWH.

Our results revealed a higher prevalence of UoHCV among MSM than among PWID (25.9% vs.7.3%, p = 0.037) in the HCV seropositive population. Since the 1990s, several measures have been implemented to promote HCV testing and to enhance the awareness of HCV infection status among PWID. These measures have achieved significant effects in increasing HCV status awareness [18, 38, 39]. The risk of sexual transmission of HCV was low in the 1990s [40]. However, since the mid-2000s, sexually transmitted HCV has been increasingly detected among sexually active MSM [6, 20]. The high prevalence of UoHCV among MSM may be attributable to their unawareness of their HCV seropositivity status due to the fundamental misunderstanding that HCV can only be contracted from intravenous exposure to infected blood (e.g., through the use of unsterile injection equipment and contaminated blood products). This explanation is supported by the finding that individuals in this study who believed that intravenous injection was a requirement for contracting HCV were more likely to have UoHCV. Furthermore, an increasing trend of MSM was observed in the HCV-seropositive PLWH group (28.3%, 68.0%, and 81.3% in periods 1, 2, and 3, respectively [p for trend < 0.001]; S2 Table), which is consistent with findings of another study conducted in Taiwan [41]. Therefore, substantial efforts are urgently required to implement interventions that reduce the prevalence rate of UoHCV among HIV-positive individuals with sexually transmitted HCV.

The lower prevalence of UoHCV among HCV-seropositive PLWH diagnosed in 2014–2020 compared with the prevalence among those diagnosed before 2008 may be attributable to the results of recent efforts to fight the spread of HCV. These efforts include the increase in publicity on HCV worldwide, newly available DAAs, and implementation of measures to ensure the affordability of oral DAA agents in Taiwan; such measures include national programs for using DAA agents to treat patients with advanced HCV (implemented in 2017–2018) and for providing treatment support to all eligible patients enrolled in Taiwan’s National Health Insurance program (2019–present) [42, 43]. This means that compared with before 2008, there is now a higher likelihood of primary physicians testing for HCV and identifying HCV seropositivity in people recently diagnosed as having HIV and informing them of their condition. Moreover, primary physicians may be more likely to inform individuals of their HCV-seropositive status when diagnosing them as having a coinfection with sexually transmitted HAV because of the emerging trend of concomitant sexually transmitted HAV and HCV among PLWH in Taiwan [41, 44].

Reducing the engagement in high-risk behavior is critical for reducing rates of HCV infection. People who have received treatment for HCV who have ongoing exposure to HCV are at risk of reinfection. Limiting such exposure is necessary for making continual progress toward eradication of the disease [45]. Studies have revealed that awareness of HCV infection status reduces engagement in high-risk behaviors among HCV-seropositive PWID [9–12]. However, a significant association was not observed between UoHCV and high-risk behaviors related to HCV infection in the present study, in which the majority (94.8%) of participants had sexually transmitted HIV infection. This finding has two possible explanations. First, individuals who were aware of their HCV status may not have known its transmission route (e.g., sexual transmission). Second, individuals may have known the transmission route but been unwilling or unable to alter their behavior accordingly. Our findings support the second explanation because the participants with awareness of their HCV-seropositive status had higher scores in knowledge domain 1 (route of HCV transmission) than those without awareness of their HCV-seropositive status, indicating that they did not alter their behavior despite knowing the HCV transmission route. The results of a cross-sectional online survey of 48 Australian MSM further support our findings. The survey revealed that most participants knew that HCV infection can be sexually transmitted between men. However, participants generally did not know how to prevent the sexual transmission of HCV [46]. Although notifying PWID of their HCV status can effectively reduce their engagement in high-risk behaviors [9–12], effective behavior modification approaches for reducing the sexual transmission of HCV in at-risk populations have yet to be identified. Moreover, interventions to reduce modifiable behavioral risk factors, such as condom distribution, the promotion of abstinence from illicit drugs, and advocacy for safe sex, may attract serious criticism because of the stigma associated with sex and drug use [47]. Therefore, to optimize the awareness of the HCV care cascade among HCV-seropositive PLWH [8], further research is necessary to identify effective interventions for modifiable behavioral risk factors and settings during the process of notifying patients of their HCV status to promote long-term reductions in behaviors that involve a high-risk of exposure to HCV [48].

In the HCV-seronegative population, the prevalence of UoHCV (61.0%) among PLWH reported in the present study is substantially higher than the prevalence of general population reported by a nationwide screening program in Taiwan (33.0%) [16], possibly because of the high proportion of MSM (76.6%) who believe that intravenous injection is a requirement for contracting HCV.

In the present study, only 288 (39.2%) of the 734 HCV-seronegative PLWH responded “yes” to the question “has your doctor ever provided information regarding HCV infection?” This number was unexpectedly low considering that these PLWH were undergoing follow-up every 1–3 months at HIV referral hospitals in Taiwan. According to Taiwan’s guidelines for HIV and acquired immunodeficiency syndrome, HCV-seronegative PLWH should undergo annual screening for HCV antibodies [49]. HIV experts may overlook the importance of informing PLWH of their HCV-seronegative status. However, our data revealed that UoHCV is associated with lower mean scores for total and domain-specific HCV-related knowledge compared with HCV status awareness among PLWH with HCV-seronegative status. Furthermore, young, sexually active PLWH may primarily consult and receive serostatus notifications of HCV infection from HIV experts because of the stigma and discrimination that PLWH may experience when consulting with non-HIV experts [50]. Therefore, HIV experts should notify sexually active PLWH of their HCV-seronegative status. This is especially crucial for individuals who believe that HCV can only be transmitted between PWID or who have been diagnosed as having a sexually transmitted disease within the preceding 6 months.

Overall, our study revealed that the correct response rate for questions on HCV-related knowledge varied considerably (16.8% to 71.1%). This finding provides a basis for targeting gaps in patient HCV knowledge during counseling. In our study, UoHCV was determined to be negatively associated with different aspects of HCV-related knowledge, which is considered critical for initiating treatment for HCV infection [28, 51]. The findings of the present study also indicate that interventions tailored to each patient’s HCV serostatus should be actively enforced to reduce the prevalence of UoHCV.

The present study has several strengths. This is the first study to analyze UoHCV among PLWH and identify different determinants associated with UoHCV among PLWH according to their HCV serostatus. The study results also aid in the customization of strategies according to their HCV serostatus for reducing the prevalence of UoHCV among PLWH. Second, no standardized, validated HCV knowledge scoring system for PLWH is currently available. This study provides clinically relevant, structural, and valid measurements of HCV-related knowledge among PLWH. This structural measure of HCV-related knowledge can be applied to assess HCV-related knowledge before and after HCV-related educational interventions. These findings can also be used to longitudinally assess the influence of knowledge regarding HCV transmission on patients’ engagement in high-risk behaviors associated with HCV infection after they have completed treatment interventions. However, this study also has several limitations. First, 525 patients were unwilling to participate, which might have caused selection bias. However, the demographic characteristics, HCV serostatus, and HIV-related risks did not differ between the enrolled and unwilling patients. Second, although the trained investigators instructed participants to recall their experiences to answer certain questions, inaccurate recall was unavoidable. Third, although the current study revealed no association between high-risk behaviors for HCV infection and UoHCV among HCV-seropositive and HCV-seronegative PLWH, this study had a cross-sectional design. Therefore, additional prospective cohort studies are warranted to further clarify changes in individuals’ engagement in high-risk behaviors after they have been notified of their HCV status. Finally, the participants in the present study were PLWH, most of whom were MSMs. Therefore, the findings may not be generalizable to all PLWH populations.

## Conclusion

With the current availability of all-oral, interferon-free DAAs, which achieve a curative outcome in more than 90% of patients with HCV and require only 12 weeks of treatment, the eradication of HCV in prevalent areas is now possible [52]; however, our study revealed that the majority of PLWH in Taiwan were unaware of their HCV serostatus. In the present study, UoHCV was associated with lower scores in different domains of HCV-related knowledge, which is considered critical for initiating treatment for HCV infection and reducing the engagement in risky behaviors among affected individuals. This finding suggests that interventions should be implemented to reduce the prevalence of UoHCV, regardless of patients’ HCV serostatus. Because the factors associated with UoHCV differed between HCV-seronegative and HCV-seropositive PLWH, strategies for reducing UoHCV may be more effective if they target PLWH according to their HCV serostastus.

## Supporting information

S1 File Questionnaire on HCV infection status and related knowledge among patients living with HIV. (DOCX)

S1 Table Validation of the original 15 questions on HCV knowledge. (DOCX)

S2 Table Trends of various HIV at-risk populations among HCV-seropositive patients living with HIV across three periods of HIV diagnosis. (DOCX)

## References

[1] Petruzziello A, Marigliano S, Loquercio G, Cozzolino A, Cacciapuoti C. Global epidemiology of hepatitis C virus infection: An up-date of the distribution and circulation of hepatitis C virus genotypes. World J Gastroenterol. 2016;22:7824–7840. 10.3748/wjg.v22.i34.7824 27678366 PMC5016383

[2] Basyte-Bacevice V, Kupcinskas J. Evolution and Revolution of Hepatitis C Management: From Non-A, Non-B Hepatitis Toward Global Elimination. Dig Dis. 2020:1–6. 10.1159/000505434 31905358

[3] The Lancet Gastroenterology H. Drug pricing: still a barrier to elimination of HCV. Lancet Gastroenterol Hepatol. 2018;3:813. 10.1016/S2468-1253(18)30354-6 30507462

[4] Aleman S, Soderholm J. Frequent loss to follow-up after diagnosis of hepatitis C virus infection: A barrier toward the elimination of hepatitis C virus. Liver Int. 2020;40:1832–1840. 32294288 10.1111/liv.14469

[5] Valencia J, Alvaro-Meca A, Troya J, Cuevas G, Gutiérrez J, Morro A, et al. High rates of early HCV reinfection after DAA treatment in people with recent drug use attended at mobile harm reduction units. Int J Drug Policy. 2019; 72:181–188. 10.1016/j.drugpo.2019.06.016 31253391

[6] Nijmeijer BM, Koopsen J, Schinkel J, Prins M, Geijtenbeek TB. Sexually transmitted hepatitis C virus infections: current trends, and recent advances in understanding the spread in men who have sex with men. J Int AIDS Soc 2019;22 (Suppl 6):e25348. 31468692 10.1002/jia2.25348 PMC6715947

[7] World Health Organization: Global Hepatitis Report 2017. 2017. (accessed on November.11th, 2020).

[8] Yehia BR, Schranz AJ, Umscheid CA, Lo Re V 3rd. The treatment cascade for chronic hepatitis C virus infection in the United States: a systematic review and meta-analysis. PLoS one. 2014;9:e101554. 10.1371/journal.pone.0101554 24988388 PMC4079454

[9] Spelman T, Morris MD, Zang G, Rice T, Page K, Maher L, et al. A longitudinal study of hepatitis C virus testing and infection status notification on behaviour change in people who inject drugs. J Epidemiol Community Health. 2015;69:745–752. 10.1136/jech-2014-205224 25814695 PMC4515217

[10] Tsui JI, Vittinghoff E, Hahn JA, Evans JL, Davidson PJ, Page K. Risk behaviors after hepatitis C virus seroconversion in young injection drug users in San Francisco. Drug Alcohol Depend. 2009;105:160–163. 10.1016/j.drugalcdep.2009.05.022 19647375 PMC2849721

[11] Kwiatkowski CF, Fortuin Corsi K, Booth RE. The association between knowledge of hepatitis C virus status and risk behaviors in injection drug users. Addiction. 2002;97:1289–1294. 10.1046/j.1360-0443.2002.00208.x 12359033

[12] Bruneau J, Zang G, Abrahamowicz M, Jutras-Aswad D, Daniel M, Roy E. Sustained drug use changes after hepatitis C screening and counseling among recently infected persons who inject drugs: a longitudinal study. Clin Infect Dis. 2014;58:755–761. 10.1093/cid/cit938 24363333

[13] Hepatitis C Guidance 2018 Update: AASLD-IDSA Recommendations for Testing, Managing, and Treating Hepatitis C Virus Infection. Clin Infect Dis. 2018;67:1477–1492. 10.1093/cid/ciy585 30215672 PMC7190892

[14] Andriulli A, Stroffolini T, Mariano A, Valvano MR, Grattagliano I, Ippolito AM, et al. Declining prevalence and increasing awareness of HCV infection in Italy: A population-based survey in five metropolitan areas. Eur J Intern Med. 2018;53:79–84. 10.1016/j.ejim.2018.02.015 29475770

[15] Cheng PN, Chiu YC, Chiu HC, Chien SC. The characteristics of residents with unawareness of hepatitis C virus infection in community. PLoS one. 2018;13:e0193251. 10.1371/journal.pone.0193251 29470547 PMC5823433

[16] Lin C, Clark R, Tu P, Tu R, Hsu YJ, Nien HC. The disconnect in hepatitis screening: participation rates, awareness of infection status, and treatment-seeking behavior. J Glob Health. 2019;9:010426. 10.7189/jogh.09.010426 31073398 PMC6486119

[17] Kuo YH, Chen PF, Wang JH, Chang KC, Kee KM, Tsai MC, et al. Comparison Stratagems of Post-Screening Management of Anti-HCV-Positive Community Residents: Simple Notification, Active Referral, or Accessible Medical Care. PLoS one. 2015;10:e0126031. 10.1371/journal.pone.0126031 25970487 PMC4430481

[18] Zhou K, Terrault NA. Gaps in Viral Hepatitis Awareness in the United States in a Population-based Study. Clin Gastroenterol Hepatol. 2020;18:188–195.e184. 10.1016/j.cgh.2019.05.047 31173892 PMC8028744

[19] Hachey DM, Holmes JT, Aubuchon-Endsley NL. Hepatitis C Treatment Cascade in a Federally Qualified Health Center. J Community Health. 2020;45:264–268. 10.1007/s10900-019-00736-4 31512110

[20] Chan DP, Sun HY, Wong HT, Lee SS, Hung CC. Sexually acquired hepatitis C virus infection: a review. Int J Infect Dis. 2016;49:47–58. 10.1016/j.ijid.2016.05.030 27270138

[21] Page K, Evans JL, Hahn JA, Vickerman P, Shiboski S, Morris MD. HCV incidence is associated with injecting partner age and HCV serostatus mixing in young adults who inject drugs in San Francisco. PLoS One. 2019;14:e0226166. 10.1371/journal.pone.0226166 31821365 PMC6903751

[22] Patel EU, Solomon SS, McFall AM, Srikrishnan AK, Pradeep A, Nandagopal P, et al. Hepatitis C care continuum and associated barriers among people who inject drugs in Chennai, India. nt J Drug Policy. 2018;57:51–60. 10.1016/j.drugpo.2018.03.023 29679811 PMC5994190

[23] Grebely J, Genoway KA, Raffa JD, Dhadwal G, Rajan T, Showler G, et al. Barriers associated with the treatment of hepatitis C virus infection among illicit drug users. Drug Alcohol Depend. 2008;93:141–147. 10.1016/j.drugalcdep.2007.09.008 17997050

[24] Fishbein DA, Lo Y, Reinus JF, Gourevitch MN, Klein RS. Factors associated with successful referral for clinical care of drug users with chronic hepatitis C who have or are at risk for HIV infection. J Acquir Immune Defic Syndr. 2004;37:1367–1375. 10.1097/01.qai.0000131932.21612.49 15483466

[25] Tsui JI, Bangsberg DR, Ragland K, Hall CS, Riley ED. The impact of chronic hepatitis C on health-related quality of life in homeless and marginally housed individuals with HIV. AIDS Behav. 2007;11:603–610. 10.1007/s10461-006-9157-8 17028996

[26] Deng LP, Gui XE, Zhang YX, Gao SC, Yang RR. Impact of human immunodeficiency virus infection on the course of hepatitis C virus infection: a meta-analysis. World J Gastroenterol. 2009;15:996–1003. 10.3748/wjg.15.996 19248201 PMC2653408

[27] Panel on Antiretroviral Guidelines for Adults and Adolescents. Guidelines for the Use of Antiretroviral Agents in Adults and Adolescents Living with HIV. 2021. Department of Health and Human Services. (accessed on April 11th, 2021)

[28] Mah A, Hull MW, DeBeck K, Milloy MJ, Dobrer S, Nosova E, et al. Knowledge of hepatitis C and treatment willingness amongst people who inject drugs in an era of direct acting antivirals. Int J Drug Policy. 2017;47:137–143. 10.1016/j.drugpo.2017.02.006 28347636 PMC5592129

[29] Treloar C, Hull P, Dore GJ, Grebely J. Knowledge and barriers associated with assessment and treatment for hepatitis C virus infection among people who inject drugs. Drug Alcohol Rev. 2012;31:918–924. 10.1111/j.1465-3362.2012.00468.x 22612899

[30] Lambers FA, Prins M, Davidovich U, Stolte IG. High awareness of hepatitis C virus (HCV) but limited knowledge of HCV complications among HIV-positive and HIV-negative men who have sex with men. AIDS care. 2014;26:416–424. 10.1080/09540121.2013.832721 24024525

[31] Yang JF, Lin CI, Huang JF, Dai CY, Lin WY, Ho CK, et al. Viral hepatitis infections in southern Taiwan: a multicenter community-based study. Kaohsiung J Med Sci. 2010;26:461–469. 10.1016/S1607-551X(10)70073-5 20837342 PMC11916219

[32] Chen CH, Yang PM, Huang GT, Lee HS, Sung JL, Sung JL, et al. Estimation of seroprevalence of hepatitis B virus and hepatitis C virus in Taiwan from a large-scale survey of free hepatitis screening participants. J Formos Med Assoc. 2007;106:148–155. 10.1016/S0929-6646(09)60231-X 17339159

[33] Denniston MM, Klevens RM, McQuillan GM, Jiles RB. Awareness of infection, knowledge of hepatitis C, and medical follow-up among individuals testing positive for hepatitis C: National Health and Nutrition Examination Survey 2001–2008. Hepatology. 2012;55:1652–1661. 10.1002/hep.25556 22213025 PMC4586034

[34] Brener L, Murphy DA, Cama EJ, Ellard J. Hepatitis C risk factors, attitudes and knowledge among HIV-positive, HIV-negative and HIV-untested gay and bisexual men in Australia. Sexual health. 2015;12:411–417. 10.1071/SH14239 26144754

[35] Balfour L, Kowal J, Corace KM, Tasca GA, Krysanski V, Cooper CL, et al. Increasing public awareness about hepatitis C: development and validation of the brief hepatitis C knowledge scale. Scand J Caring Sci. 2009;23:801–808. 10.1111/j.1471-6712.2008.00668.x 19500309

[36] Huang YF, Yang JY, Nelson KE, Kuo HS, Lew-Ting CY, Yang CH, et al. Changes in HIV incidence among people who inject drugs in Taiwan following introduction of a harm reduction program: a study of two cohorts. PLoS Med. 2014;11:e1001625. 10.1371/journal.pmed.1001625 24714449 PMC3979649

[37] Vanhommerig JW, Lambers FA, Schinkel J, Geskus RB, Arends JE, van de Laar TJ, et al. Risk Factors for Sexual Transmission of Hepatitis C Virus Among Human Immunodeficiency Virus-Infected Men Who Have Sex With Men: A Case-Control Study. Open Forum Infect Dis. 2015;2:ofv115. 10.1093/ofid/ofv115 26634219 PMC4665384

[38] Bao Y, Larney S, Peacock A, Colledge S, Grebely J, Hickman M, et al. Prevalence of HIV, HCV and HBV infection and sociodemographic characteristics of people who inject drugs in China: A systematic review and meta-analysis. Int J Drug Policy. 2019;70:87–933. 10.1016/j.drugpo.2019.05.005 31125802 PMC6679982

[39] Yen YF, Yen MY, Su LW, Li LH, Chuang P, Jiang XR, et al. Prevalences and associated risk factors of HCV/HIV co-infection and HCV mono-infection among injecting drug users in a methadone maintenance treatment program in Taipei, Taiwan. BMC public health. 2012;12:1066. 10.1186/1471-2458-12-1066 23227904 PMC3534443

[40] Feldman JG, Minkoff H, Landesman S, Dehovitz J. Heterosexual transmission of hepatitis C, hepatitis B, and HIV-1 in a sample of inner city women. Sex Transm Dis. 2000;27:338–342. 10.1097/00007435-200007000-00007 10907909

[41] Sun HY, Chang SY, Yang ZY, Lu CL, Wu H, Yeh CC, et al. Recent hepatitis C virus infections in HIV-infected patients in Taiwan: incidence and risk factors. J Clin Microbiol. 2012;50:781–787. 10.1128/JCM.06014-11 22189113 PMC3295121

[42] Administration NHI. Hepatitis C New Oral Drug. 2018; https://www.nhi.gov.tw/Content_List.aspx?n=A4EFF6CD1C4891CA&topn=3FC7D09599D2597. (accessed on November 11th, 2020).

[43] Jorgensen C, Carnes CA, Downs A. "Know More Hepatitis:" CDC’s National Education Campaign to Increase Hepatitis C Testing Among People Born Between 1945 and 1965. Public Health Rep. 2016;131 (Suppl 2):29–34. 10.1177/00333549161310S205 27168658 PMC4853325

[44] Cheng CY, Wu HH, Zou H, Lo YC. Epidemiological characteristics and associated factors of acute hepatitis A outbreak among HIV-coinfected men who have sex with men in Taiwan, June 2015-December 2016. J Viral Hepat. 2018;25:1208–1215. 10.1111/jvh.12926 29741291

[45] Martin NK, Thornton A, Hickman M, Sabin C, Nelson M, Cooke GS, et al. Can Hepatitis C Virus (HCV) Direct-Acting Antiviral Treatment as Prevention Reverse the HCV Epidemic Among Men Who Have Sex With Men in the United Kingdom? Epidemiological and Modeling Insights. Clin Infect Dis. 2016;62:1072–1080. 10.1093/cid/ciw075 26908813 PMC4826456

[46] Hopwood M, Lea T, Aggleton P. Multiple strategies are required to address the information and support needs of gay and bisexual men with hepatitis C in Australia. J Public Health. 2016;38:156–162. 10.1093/pubmed/fdv002 25626415

[47] Lee CY, Wu PH, Tsai JJ, Chen TC, Chang K, Lu PL. Cascade Analysis of Anonymous Voluntary HIV Counseling and Testing Among Patients with HIV Infection in Taiwan. AIDS Patient Care STDS. 2020;34:303–315. 10.1089/apc.2020.0044 32639210

[48] Hagan H, Neurer J, Jordan AE, Des Jarlais DC, Wu J, Dombrowski K, et al. Hepatitis C virus infection among HIV-positive men who have sex with men: protocol for a systematic review and meta-analysis. Syst Rev. 2014;3:31. 10.1186/2046-4053-3-31 24669911 PMC3986916

[49] Centers for Disease Control MoHaW, R.O.C.(Taiwan) Guidelines for Diagnosis and Treatment of HIVAIDS (4E). 2013; Centers for Disease Control, Ministry of Health and Welfare, R.O.C.(Taiwan).

[50] Parker R, Aggleton P. HIV and AIDS-related stigma and discrimination: a conceptual framework and implications for action. Soc Sci Med. 2003;57:13–24. 10.1016/s0277-9536(02)00304-0 12753813

[51] Zeremski M, Dimova RB, Zavala R, Kritz S, Lin M, Smith BD, et al. Hepatitis C virus-related knowledge and willingness to receive treatment among patients on methadone maintenance. J Addict Med. 2014;8:249–257. 10.1097/ADM.0000000000000041 24820257 PMC4747636

[52] Asselah T, Boyer N, Saadoun D, Martinot-Peignoux M, Marcellin P. Direct-acting antivirals for the treatment of hepatitis C virus infection: optimizing current IFN-free treatment and future perspectives. Liver Int. 2016;36 Suppl 1:47–57. 10.1111/liv.13027 26725897

