# Supplementary material for: High prevalence of unawareness of HCV infection status among both HCV-seronegative and seropositive people living with human immunodeficiency virus in Taiwan
Source: PLoS One. 2021 May 6;16(5):e0251158. doi: 10.1371/journal.pone.0251158 (PMC8101914; doi:10.1371/journal.pone.0251158)
Supplement: S1 File — (DOCX) [file pone.0251158.s001.docx]

**S1 File**

**Questionnaire on HCV infection status and related knowledge among patients living with HIV**

**Part: Sociodemographic characteristics**

- 1. Date of birth: (year) (month) (date)
  2. Date of HIV test: (year) (month) (date)
  3. Biological sex: male ; female
  4. Highest education level: none___; elementary school ; junior high school ; senior high school ; junior college ; bachelor’s degree ; master’s degree ; doctoral degree
  5. Occupation: student ; military personnel ; government employee or civil servant ; employee ; professional ; unemployed ; other (please specify)
  6. Marital status: single ; married ; separated ; divorced ; widowed ; domestic partnership
  7. Chronic comorbid disease: diabetes___; hypertension___; cardiac disease___; kidney disease___; asthma___; tuberculosis___; cancer___; depression___; bipolar disorder___; insomnia___; chronic hepatitis B___; chronic hepatitis C___; none of the above___
  8. Chronic medication: hypnotics___; neurological medicine (antidepressants, agrypnotics, lithium) ___; Chinese medicine___; others___; none___
  9. Sexual orientation：homosexual ; bisexual ; heterosexual
  10. Sex role：bottom (0)___; top (1)___; versatile___
  11. Reason for infection: sexual activities ; intravenous drug user (MDMA not included) ; blood transfusion ; vertical transmitted infection ; hemophilia___; uncertain

1.12 Have you ever have a hstory of sexually-transmitted diseases within the

preceding 6 months.

**Part: Self-awareness of hepatitis C virus conditions. (This part is mainly to know your self-awareness of hepatitis C virus infection status.)**

1. Have you ever heard of hepatitis C virus ? Yes__; No__.

2. Has the doctor who cared for you mentioned information on hepatitis C virus to you before? Yes__; No__; I do not known__.

3. Do you know your own hepatitis C virus infection status? Yes__; No__. (If you answered “No”, please skip to “**Part: HCV knowledge**”.)

4. What are your hepatitis C virus infection conditions? Positive__; Negative__. (If you are negative, please skip to “**Part: HCV knowledge**”.)

5. Has your doctor ever told you of hepatitis C virus treatments? Yes__; No__; I do not known__. (If you answered “No”, please skip to “**Part: HCV knowledge**”.)

6. Have you accepted treatments for hepatitis C virus? Yes__; No__. (If you answered “Yes”, please skip to “**7. What are your current treatments?**”)

A. What is your reason for not accepting treatments?

- I do not think it is necessary__
- The side effects are too bad__
- The treatment effects are bad__
- The treatments are inconvenient__
- Other__.

(Multiple choices are accepted, and please skip to “Part: HCV knowledge” after answering.)

7. What are your current treatments?

- Interferon + ribavirin__
- Oral antihepatitis C virus medications__.

8. What are the results of your treatments? Success__; treatment interrupted__; treatment failure__. (If you answered “success” or “treatment interrupted”, please skip to “**Part: HCV knowledge**”.)

9. Why did treatment fail?

- Treated with interferon and ribavirin for 12 weeks but medications stopped because early virological response not achieved__
- Treatments completed but viruses still detected__
- After treatments were completed and no viruses detected, viruses detected again after stopping medications for 24 weeks__.

**Part: HCV knowledge. (This part is mainly to know your knowledge of HCV infection.)**

1. Does hepatitis C virus can be transmitted through the blood?

Yes__; No__; I do not know__.

2. Does hepatitis C virus can be transmitted through sexual behaviors?

Yes__; No__; I do not know __.

3. Does hepatitis C virus can be transmitted through mother-to-child vertical

transmission?

Yes__; No__; I do not know __.

4. Are the infection routes of HIV similar to those of the hepatitis C virus?

Yes__; No__; I do not know __.

5. If you are infected with HIV, does this mean you are more likely to be infected

with hepatitis C virus?

Yes__; No__; I do not know __.

6. Does the successful treatment of hepatitis C virus infection prevent reinfection?

Yes__; No__; I do not know __.

7. During sexual behaviors, does mucosa hemorrhage of sexual contact parts due

to excessive intensity makes hepatitis C virus infection easier?

Yes__; No__; I do not know __.

8. Is blood the major transmission routes of hepatitis C virus?

Yes__; No__; I do not know __.

9. Hepatitis C virus mostly cures itself, and no treatment is needed?

Yes__; No__; I do not know __.

10. Does Hepatitis C virus infection commonly not result in any symptoms?

Yes__; No__; I do not know __.

11. Do complications after hepatitis C virus infection include cirrhosis and liver

cancer?

Yes__; No__; I do not know __.

12. Does HIV increase complication probability after hepatitis C virus infection

(such as cirrhosis and liver cancer)?

Yes__; No__; I do not know __.

13. Can hepatitis C virus infection be prevented by vaccines?

Yes__; No__; I do not know __.

14. Can hepatitis C virus infection be treated?

Yes__; No__; I do not know __.

15. Can Hepatitis C virus infection be cured?

Yes__; No__; I do not know __.

**Part: Perceived risk of HCV infection. (This part is mainly to know your perceived risk of HCV infection.)**

|  | Strongly disagree | Disagree | Neither agree nor disagree | Agree | Strongly agree |
| --- | --- | --- | --- | --- | --- |
| Only those who inject medication intravenously can get hepatitis C |  |  |  |  |  |
| The sexual behavior styles that I like put me at risk of hepatitis C infection. |  |  |  |  |  |
| I am more worried about hepatitis C virus than HIV. |  |  |  |  |  |

**Part: Assessment of potential exposures to HCV within the preceding 6 months: Sexual behavior. (This part is mainly to know whether you have had a risk of hepatitis C virus infection from sexual behavior within the preceding 6 months.)**

1. Have you engaged in sexual behavior with “fixed” partners within the preceding 6 months? Yes__; No__. (If you answered “No”, please skip to question “5”.)

2. How would you describe your partners? Homosexual__; Bisexual__; Heterosexual__; Transsexual__.

3. How often have you worn condoms during vaginal intercourse with your partners within the preceding 6 months? Never__; Sometimes__; Always__; Did not have vaginal intercourse with partners.

4. How often have you worn condoms during anal intercourse (including contact of the anus with mouths, hands, and genitals) with your partners within the preceding 6 months? Never__; Sometimes__; Always__; Did not have anal intercourse with partners. (Please skip to question 11 after answering.)

5. Have you engaged in sexual behavior with casual partners within the preceding 6 months? Yes__; No__. (If you answered “No”, please skip to “HCV infection risk evaluation [past 6 months; vein medication injection]”.)

6. How did you know or contact your casual partners? Through the Internet__; In a club__; In a bar__; Introduced through friends__; In a sauna__; In a park__; Through a mobile phone application__; At a home party__; Prostitution__; Other__. (Multiple choices are accepted.)

7. How would you describe your partners? Homosexual__; Bisexual__; Heterosexual__; Transsexual. (Multiple choices are accepted.)

8. How many different people have you engaged in sexual behavior with within the preceding 6 months? 1__; 2–5__; 6–10__; 11–20__; 21–50__; over 50__.

9. How often have you worn condoms during vaginal intercourse with your partners within the preceding 6 months? Never__; Sometimes__; Always__; Did not have vaginal intercourse with partners.

10. How often have you worn condoms during anal intercourse (including contact of the anus with mouths, hands, and genitals) with your partners within the preceding 6 months? Never__; Sometimes__; Always__; Did not have anal intercourse with partners__.

11. Why have you not used condoms within the preceding 6 months? (Multiple choices are accepted.)

No condoms were available__; Condoms are too expensive__; Sexual partners refused to use them__; Other birth control methods were used__; I did not think it was necessary__; It did not occur to me__; I am not comfortable using them__; Other__.

12. How often have you engaged in the following behavior with your sexual partners within the preceding 6 months?

|  | Never | Ever | | |
| --- | --- | --- | --- | --- |
|  |  | Sometimes | Often | Always |
| Using sex toys during sexual behavior. |  |  |  |  |
| Having sadomasochism (SM) type sexual behavior and bleeding in the process. |  |  |  |  |
| Having SM type sexual behavior and not bleeding in the process. |  |  |  |  |
| Group sexual behavior (more than two people, male and female). |  |  |  |  |
| Group sexual behavior (more than two people, only male). |  |  |  |  |
| Group sexual behavior (more than two people, only female). |  |  |  |  |
| Mouth-anal intercourse, inserter. |  |  |  |  |
| Mouth-anal intercourse, insertee. |  |  |  |  |
| Finger-anal intercourse, inserter. |  |  |  |  |
| Finger-anal intercourse, insertee. |  |  |  |  |
| Fist-anal intercourse, inserter. |  |  |  |  |
| Fist-anal intercourse, insertee. |  |  |  |  |
| Vaginal intercourse. |  |  |  |  |

13. Have you ever cleaned the anus before anal intercourse with partners in the preceding

6 months? Never__; Sometimes__; Often__; Always. (If you answered

“Never”, please answer question 15.)

14. What is the cleaning method you have adopted? (Multiple choices are accepted.)

Anal douching__; Shower head entering the anus for cleansing__; Surface (not

entering the anus) cleansing__; Other__.

15. Did you use sexualized drug during sexual behavior within the preceding 6 months?

Yes__; No__. (If you answered “No”, please skip to **Part: Risk evaluation of**

**hepatitis C virus infection within the preceding 6 months: medication injection.**)

15.1 How often (on average per month) have you used sexualized drug within the preceding 6 months? Over 28 times per month __; 24–28 times per month__; 16–24 times per month__; 8–16 times per month__; 4–8 times per month__; 2–4 times per month__; 1–2 times per month__; Other__.

15.2 Which sexualized drug have you used within the preceding 6 months? (Multiple choices are accepted.)

Heroin __; Cocaine__; Crack__; Amphetamine__; MDMA__; Marijuana__; Morphine__; Secobarbital__; Amobarbital__; Methaqualone__; FM2__; Ketamine__; LSD__; Erimine__; Methadone (not prescribed from hospital)__; Methamphetamine__; GHB__; Black-cat__; RUSH__; 5-meo__; G-spot gel__; Mephedrone__; Other__.

15.3 What was the method of using sexualized drug within the preceding 6 months?

|  | Yes | No |
| --- | --- | --- |
| Injection |  |  |
| Sniffing |  |  |
| Oral |  |  |
| Anal |  |  |

15.4 Have you ever used condoms during sexual behavior accompanied with drug

use? Never__; Sometimes__; Always.

15.4.1 From the previous question, what was the reason for not using condoms? (Multiple choices are accepted.)

It did not occur to me because I was high__; Sexual partners refused to use them __; I did not think it was necessary__; They happened to not be around me__; I was not comfortable using them __; Other__.

15.5 Have you ever gone to a mental clinic for abstinence treatment within the preceding 6 months? Yes__; No__.

**Part: Assessment of the risk of HCV infections within the preceding 6 months: Recreational drug injection. (This part is mainly to know whether you have a risk of hepatitis C virus infection from injecting recreational drugs.)**

1. Have you (including now) ever experienced injecting recreational drugs? Yes__; No__. (If you answered “No”, please stop.)

1.1 How long have you been injecting recreational drugs? Less than 1 year__; 1–2 years__; 2–3 years__; over 3 years.

2. Have you (including now) ever experienced injecting recreational drugs within the preceding 6 months? Yes__; No__. (If you answered “No”, stop answer)

2.1 How often (on average per month) have you injected recreational drugs within the preceding 6 months? 28 times per month__; 24–28 times per month__; 16–24 times per month__; 8–16 times per month__; 4–8 times per month__; 2–4 times per month__; 1–2 times per month__; Other__.

2.2 Which recreational drug have you injected? (Multiple choices are accepted.)

Heroin__; Morphine__; Opium__; Cocaine__; Crack__; Amphetamine__; Pentazocine__; FM-2__; Stilnox__; Other__.

2.3 Have you shared injection equipment with others when injecting recreational drugs within the preceding 6 months?

Yes__; No__. (If you answered “No”, please skip to 2.4.)

2.3.1 What injection equipment have you shared?

|  | Yes | No |
| --- | --- | --- |
| Needle |  |  |
| Syringe |  |  |
| Diluent |  |  |
| Injection container |  |  |

2.3.2 How many people have you shared injection equipment with (including needles,

syringes, diluent, and containers)? __people.

2.3.3 Do you understand the hepatitis C virus infection conditions of the people you

shared injection equipment with? Yes__; No__; I do not known.

2.4 What was the source of your diluent within the preceding 6 months? Tap water__; Distilled water__; Mineral water__; Toilet water__; Normal saline__; Other__.

2.5 Have people around you injected drugs when you were engaging in sexual behavior within the preceding 6 months? Yes__; No__; I do not known__.

2.6 Have you travelled to Southeast Asia or China within the preceding 6 months? Yes__; No__.
