# Supplementary material for: High prevalence of unawareness of HCV infection status among both HCV-seronegative and seropositive people living with human immunodeficiency virus in Taiwan
Source: PLoS One. 2021 May 6;16(5):e0251158. doi: 10.1371/journal.pone.0251158 (PMC8101914; doi:10.1371/journal.pone.0251158)
Supplement: S1 Table — (DOCX) [file pone.0251158.s002.docx]

S1 Table. Validation of the original 15 questions on HCV knowledge.

|  | *Correct answer* | *Correct rate*  *N=844, n (%)* | *Item analysis* | *Exploratory factor analysis* ^a,b^ | *Cronbach’s alpha* | *Final established domain* |
| --- | --- | --- | --- | --- | --- | --- |
| 1. Does hepatitis C virus can be transmitted through the blood? | Yes | 547 (64.8) | Good discrimination | Route of HCV transmission | Good reliability  (0.829) | Domain 1 |
| 2. Does hepatitis C virus can be transmitted through sexual behaviors? | Yes | 477 (56.5) | Good discrimination |  |  |  |
| 3. Does hepatitis C virus can be transmitted through mother-to-child vertical transmission? | Yes | 454 (53.8) | Good discrimination |  |  |  |
| 4. Are the infection routes of HIV similar to those of the hepatitis C virus? | Yes | 472 (55.9) | Good discrimination |  |  |  |
| 5. During sexual behaviors, does mucosa hemorrhage of sexual contact parts due to excessive intensity makes hepatitis C virus infection easier? | Yes | 473 (56.0) | Good discrimination |  |  |  |
| 6. Is blood the major transmission routes of hepatitis C virus? | Yes | 425 (50.4) | Good discrimination |  |  |  |
| 7. If you are infected with HIV, does this mean you are more likely to be infected with hepatitis C virus? | Yes | 439 (52.0) | Good discrimination | Course and complication of HCV | Good reliability  (0.852) | Domain 2 |
| 8. Does successful treatment of hepatitis C virus infection prevent reinfection? | No | 332 (39.3) | Good discrimination |  |  |  |
| 9. Hepatitis C virus mostly cures itself, and no treatment is needed? | No | 537 (63.6) | Good discrimination |  |  |  |
| 10. Does hepatitis C infection commonly not result in any symptoms? | Yes | 264 (31.3) | Good discrimination |  |  |  |
| 11. Do complications after hepatitis C virus infection include cirrhosis and liver cancer? | Yes | 514 (60.9) | Good discrimination |  |  |  |
| 12. Does HIV increase complication probability after hepatitis C virus infection (such as cirrhosis and liver cancer)? | Yes | 444 (52.6) | Good discrimination |  |  |  |
| 13. Can hepatitis C virus infection be prevented by vaccines? | No | 142 (16.8) | Good discrimination | Treatment of HCV | Good reliability  (0.783) | Domain 3 |
| 14. Can hepatitis C virus infection be treated? | Yes | 600 (71.1) | Good discrimination |  |  |  |
| 15. Can Hepatitis C virus infection be cured? | Yes | 387 (45.9) | Good discrimination |  |  |  |

Note:

^a^ In the exploratory factor analysis, the Kaiser–Meyer–Olkin measure of sampling adequacy was 0.949, and Bartlett’s test of sphericity was χ2 = 6170.870 (degree of freedom = 105, *P* < .001).

^b^ Factors were retained if they had an eigenvalue of >1, preceded the “elbow” in a scree plot, and if the set of items collectively accounted for 70%–80% of the variance. Factor loadings were used to determine the number of items included within each factor, and items with a factor loading of >0.4 were retained.

Abbreviations:

HCV, hepatitis C virus; HIV, human immunodeficiency virus.
