# Supplementary material for: High prevalence of unawareness of HCV infection status among both HCV-seronegative and seropositive people living with human immunodeficiency virus in Taiwan
Source: PLoS One. 2021 May 6;16(5):e0251158. doi: 10.1371/journal.pone.0251158 (PMC8101914; doi:10.1371/journal.pone.0251158)
Supplement: S2 Table — (DOCX) [file pone.0251158.s003.docx]

S2 Table. Trends of various HIV at-risk populations among HCV-seropositive patients living with HIV across three periods of HIV diagnosis

|  | All participants | Period 1 (before 2008)  N=53 | Period 2 (2008–2013)  N=25 | Period 3 (2014–2020)  N=32 | *P* for trend^a^ |
| --- | --- | --- | --- | --- | --- |
| MSM, n (%) | 58 | 15 (28.3) | 17 (68.0) | 26 (81.3) | <0.001 |
| Heterosexual, n (%) | 8 | 2 (3.8) | 3 (12.0) | 3 (9.4) | 0.282 |
| Bisexual, n (%) | 3 | 2 (3.8) | 0 (0.0) | 1 (3.1) | 0.772 |
| PWID, n (%) | 41 | 34 (64.2) | 5 (20.0) | 2 (6.3) | <0.001 |

Note:

^a^The Cochran–Armitage trend test was used to conduct trend analyses of the four dichotomized classes of HIV at-risk populations across the three periods of HIV diagnosis.

Abbreviations:

MSM, men who have sex with men; PWID, people who inject drugs.
